# Medical Teaching in New York

**Published:** 1850-10

**Authors:** 


MEDICAL TEACHING IN NEW YORK.

The New York Register of Medicine and Pharmacy indulges the belief, that recent movements in the city of New York, "will do much towards making that city, what it should be: the metropolis of medicine, as it is of other interests." We are inclined to think that the belief is more a matter of hope, than faith, and we are satisfied that it is a hope that will not soon grow into fruition. In the nature of things it is impossible that anything like centralization can flourish under the political institutions of this country, and the diversified climates, habits of the people, and peculiarities of sections, in this country, form an insurmountable barrier to centralization in medical teaching. In the contest for supremacy, the West is abundantly able to maintain that proud position in medical science, she has held for thirty years. From a long and intimate acquaintance with the subject, we unhesitatingly affirm, that in no part of the Union has the science of medicine been better taught, than in the West, and so far from having lost any of her former elements of capacity for medical teaching, we feel well assured that her teachers have kept pace, with those of the East. Her pupils are scattered over the West and South, and will compare favorably with practitioners of any school that may be named. Those who live among the people of the West, who draw their observations from actual life, and who know the modifications from an Eastern standard, necessary to be made in the treatment of the diseases of the West and South, are better prepared to teach, than those, who aspiring to teach, have first to be taught. Without wishing in the slightest degree to disparage the schools of the city of New York, we feel that we do nothing more than justice to truth in saying, that Louisville is better qualified for making successful practitioners in the South and West, than New York can possibly be. The students see in the Louisville Hospital, the modifications of disease, that are the natural product of peculiar climate and habits of living, and they are taught the means of treating these modifications by those who have experience. Hospital facilities, extensive library privileges, and the instruction of native teachers whose qualifications are ample, should equip students fully for the successful practice of their profession, and in the discharge of the duties of practice they should have as little to unlearn as possible.

But it may be urged that one of the New York schools has selected two Western professors as teachers. This, however, does not militate against the doctrine we are endeavoring to establish. The hospitals in New York are Eastern institutions; the diseases found in them vary widely, in many of their forms, from the maladies that are indigenous to the West, and the teaching must be done with classes, a large majority of whose numbers are Eastern men. The West has long ceased being much of a tributary to the medical schools of the East, and the practice of sending young men from the West, to be educated in the East, as physicians, is rapidly diminishing. It is considered almost essential that a Western lawyer shall be prepared for the practice of his profession, at home, and the reasons that require the same for the physician are quite as imperative. The forms of practice in both professions, being practiced at home in a peculiar way, are best taught at home. B.

